# Optimized Extraction by Response Surface Methodology Used for the Characterization and Quantification of Phenolic Compounds in Whole Red Grapes (*Vitis vinifera*)

**DOI:** 10.3390/nu10121931

**Published:** 2018-12-05

**Authors:** Lisard Iglesias-Carres, Anna Mas-Capdevila, Lucía Sancho-Pardo, Francisca Isabel Bravo, Miquel Mulero, Begoña Muguerza, Anna Arola-Arnal

**Affiliations:** 1 Nutrigenomics Research Group, Departament de Bioquímica i Biotecnologia, Universitat Rovira i Virgili, 43007 Tarragona, Spain; lisard.iglesias@urv.cat (L.I.-C.); anna.mas@urv.cat (A.M.-C.); lucia.sancho@estudiants.urv.cat (L.S.-P.); franciscaisabel.bravo@urv.cat (F.I.B.); miquel.mulero@urv.cat (M.M.); anna.arola@urv.cat (A.A.-A.); 2 EURECAT, Centre Tecnològic de Catalunya, Unit of nutrition and Health, 43204 Reus, Spain

**Keywords:** anthocyanin, flavanols, flavonoids, proanthocyanidins, response surface methodology

## Abstract

Scientific research has focused on the characterization of bioactive polyphenols from grape seeds and skins, and the pulp has often been overlooked. However, since the beneficial properties of grapes are associated with the consumption of whole fruit, a full extraction and posterior characterization of the phenolic compounds in whole grapes is required to identify the involved bioactive compounds. Such methodologies are not currently available for the whole edible parts of red grapes. This study aimed to determine the best polyphenol extraction conditions of whole red grapes, and apply the method to characterize and quantify the polyphenol composition of three different grapes. The optimized conditions were 80 mL/g, 65% methanol (1% formic acid), 72 °C, and 100 min under agitation of 500 rpm. Also, methanol and ethanol were compared as extraction solvents, and methanol achieved statistically higher extraction rates for anthocyanins. The results of this work suggest a higher quantification of phenolic compounds when red grapes are analyzed whole, including the seeds, pulp, and skin.

## 1. Introduction

Polyphenols are plant secondary metabolites that are found in high concentrations in a wide variety of foods. More than 8000 phenol structures have been described, and several are found only in a limited number of species. Due to their beneficial health properties [1], polyphenols have attracted a large amount of interest in recent years. Among foods, grapes are one of the fruits with the highest polyphenol content, and have been investigated by many authors [2,3,4,5]. Phenolic compounds are differentially distributed in the skins, pulp, and seeds of grapes [6]. The beneficial health effects of these compounds have been reported from the consumption of phenolic compounds from whole grapes [7], grape juice [8,9], and grape seeds [10].

Red grapes are rich in both flavonoid and non-flavonoid compounds [2,3]. Anthocyanins are the main group of polyphenols found in red grapes. Malvidin-3-*O*-glucoside is the predominant anthocyanin in red grapes, but cyanidin-3-*O*-glucoside, delphinidin-3-*O*-glucoside, petunidin-3-*O*-glucoside, and peonidin-3-*O*-glucoside also occur in considerable amounts [3,11]. Flavanols are also present in considerable quantities, and (+)-catechin and (−)-epicatechin are the most abundant forms. Several derivatives, such as epicatechin gallate, epigallocatechin gallate, epigallocatechin, and procyanidin dimer B1 and B2 have also been reported [2,3,5]. In red grapes, flavonols are less distributed than anthocyanins. Despite this, quercetin, kaempferol, and isorhamnetin glucosides can occur in different cultivars [2,3]. The most abundant phenolic acids in grapes occur as hydroxycinnamic acids, especially as tartaric conjugates [2,5]. In addition, several hydroxybenzoic acid derivates have been described in red grapes, of which gallic acid is the most abundant compound [3]. Resveratrol and its glucoside form trans-polydatin have been identified as the most common components of the stilbene group found in red grapes. However, their levels are low, and only occur in skins [3,5].

Phenolic compounds are plant stress metabolites [12,13], and several factors have an impact on the phenolic profile of fruits [2], including grapes [3,14,15]. Specifically, the cultivation method can greatly modulate the phenolic content of grapes [16]. For example, organic cultivation is known to modulate the phenolic profile of grapes [15]. Additionally, grape varieties can present markedly different phenolic profiles [4,14,17]. In the specific case of grapes, different phenolic profiles have been demonstrated to give rise to different biological effects [8,9]. For example, the consumption of two different wines with noteworthy phenolic profiles led to different low density lipoprotein (LDL)-oxidation outcomes [9]. Therefore, it is essential to characterize the food matrix in order to correlate it with certain beneficial health effects. To do so, specific extraction methods must be developed, as a multitude of factors can affect the phenolic extraction from food matrixes [18]. In this sense, the extraction temperature, extraction solvent, liquid-to-solid ratio (LSR), and extraction time have been reported to have an impact on the extraction of phenolics from grape cranes, seeds, skins, and other grape by-products [19,20,21,22,23,24]. Traditional optimization studies take into account only one factor at time, while other factors are kept constant. Consequently, this approach is expensive, laborious, and time-consuming [25]. Its main disadvantage is that interactions between variables cannot be evaluated and are overlooked [25,26]. In this sense, response surface methodology (RSM) is a useful strategy [18]. RSM enables the evaluation of the effects of different independent variable interactions between themselves and between dependent variables [25,26]. Indeed, RSM has been widely used in the optimization of phenolic compound extraction from several vegetal sources [19,25,26]. However, the one-factor-at-a-time approach is useful in the selection of the experimental ranges that are used in RSM designs [27].

Notably, phenolic compounds are abundant in grape seeds and skins, and many studies only focus on the characterization of these grape tissues [4,17,28,29]. However, grape pulp also contains phenolic compounds [5,6,30], with the main family being the hydroxycinnamic acids [31]. Although some authors have evaluated the phenolic content of whole grapes [32,33], the total polyphenol content in red grapes appears to be underestimated, as most studies usually do not include grape pulps on their studies [4,15,16,17,28]. A full profile of the edible parts of grapes, which includes the pulp, is essential to link the health benefits associated with their consumption to their phenolic profile. In this sense, hydroxycinnamic acids have been reported to have diverse health functions [34,35], which makes their contribution to the beneficial bioactivity of whole grape consumption plausible.

There are several studies that have optimized the methods to extract phenolics from grape seeds, skins, cranes, stalks, and other grape by-products [19,20,24,28,36,37]. However, studies optimizing the extraction of the major phenolic families in red grapes as a whole (including skins, pulp, and seeds) are lacking. Given that grapes are typically consumed whole, the study of the whole matrix is of key importance when linking grape consumption and beneficial health effects. Therefore, this study aimed to develop an easy-to-perform extraction method that is capable of extracting the most representative phenolics from whole red grape varieties. Additionally, this method was used to profile the phenolic content of two whole red Grenache grape cultivars—one produced organically (OG) and the other conventionally (CG)—and a Peruvian Red Globe grape (PG).

## 2. Materials and Methods

### 2.1. Plant Material

Organic (OG) and non-ecological (conventional, CG) Grenache grapes (*Vitis vinifera*) were harvested at maturity in the region of Rasquera (Tarragona, Spain). To assure that the only agronomic variable influencing the phenolic profile of the red Grenache grapes was the cultivation system, both OG and CG were harvested on the same day (26 September 2015) from contiguous vineyards. Peruvian (PG) Red Globes (*Vitis vinifera*) were purchased from Mercabarna (Barcelona, Spain). Pedicels were manually removed, and whole grapes, which included skins, seeds, and pulp, were frozen in liquid nitrogen and later ground to homogeneity. Next, the homogenates were lyophilized for one week in a Telstar LyoQuest lyophilizer (Thermo Fisher Scientific, Madrid, Spain) at −85 °C. The lyophilized homogenates were ground to a fine powder. The grape powder was kept dry and protected from humidity and light exposure until extraction. OG was used in the optimization studies, while CG and PG were used to provide insights into the different phenolic compositions in different fruit varieties and cultivation methods.

### 2.2. Chemicals and Reagents

Acetonitrile, methanol, ethanol (HPLC analytical grade), and glacial acetic acid were purchased from Panreac (Barcelona, Spain). Formic acid was purchased from Scharlab (Barcelona, Spain). Ultrapure water was obtained from a Milli-Q Advantage A10 system (Madrid, Spain). The Folin–Ciocalteu and p-dimethylaminocinnamaldehyde (DMACA) reagents were purchased from Fluka/Sigma-Aldrich (Madrid, Spain). Gallic acid (GA), (−)-epicatechin (Ecat), p-coumaric acid (pCou), and (+)-catechin (Cat) were purchased from Fluka/Sigma-Aldrich; chlorogenic acid (Chl), malvidin-3-*O*-glucoside (Mv3G), (−)-epigallocatechin gallate (EGCG), and procyanidin dimer B2 (B2) were purchased from Extrasynthése (Lyon, France); cyanidin-3-*O*-rutinoside (Cy3R) was purchased from PhytoLab (Vestenbergsgreuth, Germany); resveratrol (Rvt) was purchased from Carl Roth (Karlsruhe, Germany); and rutin (Rut) was kindly provided by Nutrafur S.A. (Murcia, Spain).

Ecat, pCou, Cat, Chl, EGCG, B2, Rvt, and Rut were individually dissolved in methanol (MetOH) at 2000 mg/L, while Mv3G and Cy3R were dissolved in MetOH (0.01% HCl) at 500 mg/L. All standard stock solutions were freshly prepared every three months and stored in amber glass flasks at −20 °C. Mixed standard stock solutions of Ecat, p-Cou, Cat, Chl, Mv3G, EGCG, B2, Cy3R, Rvt, and Rut were prepared with water:acetic acid (95:5 *v*/*v*) to obtain the concentration needed to construct the calibration curves.

### 2.3. Polyphenol Extraction

Grape powder was weighed (0.1 g, 0.2 g, 0.4 g, and 0.8 g) to obtain the desired LSR, and was mixed with one mL of preheated extraction solvent (methanol:water, *v*:*v*). Different extraction MetOH proportions (1% formic acid), extraction temperatures, times, and extraction steps were used throughout the experiment. In all of the cases, the methanol solution included 1% formic acid. Extractions were performed at 500 rpm agitation with protection from light exposure. Once the extraction was completed, the samples were centrifuged at 9500× *g* for 10 min at 4 °C, and the supernatants were stored at −20 °C until further use.

### 2.4. Single-Factor Studies

To select the working ranges for the RSM independent variables, the effect of LSR, methanol concentration, and temperature on the extraction of grape phenolics were evaluated based on total phenolic content (TPC), total anthocyanin content (TAC), and total flavanol content (TFC) extracted from OG, as these variables represent the major phenolic families found in grapes [2,3]. The LSR was evaluated at the ratios of 10 mL/g, 20 mL/g, 40 mL/g, and 80 mL/g; temperatures of 25 °C, 40 °C, 55 °C, 70 °C, and 85 °C; and methanol proportions of 30%, 50%, 60%, 70%, and 90%. All of the extractions lasted for 30 min, and the extraction variables were kept constant at 80 mL/g, 55 °C, and 50% when not evaluated.

### 2.5. Response Surface Design

The extraction was optimized with OG using an RSM experimental design. A face-centered central composite design with two factors was selected. It consisted of 11 randomized runs, with three center-point replicates. The independent variables used in the RSM were MetOH proportion (40–80%, *X_i_*) and temperature (40–85 °C, *X_j_*). The LSR (80 mL/g) and extraction time (30 min) were fixed as constant variables during the RSM experiment. The experimental data were fitted to a second polynomial response surface, which follows Equation (1):

$$Y=\;\beta_{0}+\overset{2}{\underset{i=1}{\sum }}\;\beta_{i}X_{i}+\;\overset{2}{\underset{i=1}{\sum }}\;\beta_{ii}\;X^{2}{}_{ii}+\;\overset{\;}{\underset{i=1}{\sum }}\overset{\;}{\underset{j=i+1}{\sum }}\beta_{ij}\;X_{ii}X_{ji}$$

where *Y* is the dependent variable, *β*_0_ is the constant coefficient, and *β_i_*, *β_ii_*, and *β_ij_* are the linear, quadratic, and interaction regression coefficients, respectively. *X_i_*, *X_ii_*, and *X_ji_* represent the independent variables.

Individual phenolic compounds were quantified by the high-performance liquid chromatography with a diode array detector (HPLC-DAD) method and used in the RSM optimization study. The results of the RSM design were analyzed with Design-expert 9.0.6 software (Trial version, Stat-Ease Inc., Minneapolis, MN, USA). Single parameters that were not influenced by the extraction factors were omitted from the model.

### 2.6. Kinetic Study

A kinetic study was performed to evaluate the effect of time on the polyphenol extraction yield of OG. Seven extraction times, ranging from 0 to 120 min, were selected. The LSR was fixed at 80 mL/g, the MetOH percentage was fixed at 65%, and the temperature was fixed at 72 °C. TPC, TAC, and TFC were determined for all of the extracts and used to evaluate the effect of time on the polyphenol extractability.

### 2.7. Sequential Extractions

Three consecutive extractions were performed to evaluate the influence of multiple extractions on the polyphenol extraction yield in OG. The extractions were carried out under the following conditions: LSR of 80 mL/g, MetOH proportion of 65%, temperature of 72 °C, and extraction time of 100 min. The TPC, TAC, and TFC were determined for all of the extracts and were used to evaluate the effect of sequential extractions on the polyphenol extraction yield.

### 2.8. Application of the Method

The specific and optimized extraction methodology was used to characterize the phenolic profiles of OG, CG, and PG. In brief, the extraction conditions were as follows: LSR of 80 mL/g, MetOH or ethanol (EtOH) proportion of 65% (1% formic acid), temperature of 72 °C, and extraction time of 100 min.

### 2.9. Analysis of Response Variables

#### 2.9.1. Total Phenolic Content

The TPC of the extracts was determined by the Folin–Ciocalteu method adapted from Nenadis et al. [35]. Briefly, 10 µL of the extract and 50 µL of the Folin–Ciocalteu reagent were successively added to an Eppendorf tube containing 500 µL of Milli-Q water and mixed. The samples were kept in the dark for three minutes, and 100 µL of Na_2_CO_3_ (25%) was added. The samples were brought to a final volume of one mL with Milli-Q water and were maintained in the dark for one h. The absorbance was read at 725 nm using an Eon BioTek spectrophotometer (Izasa, Barcelona, Spain) against a water sample (blank) that underwent identical treatment. GA was used to construct the calibration curve between 40–400 mg/L. The results were expressed as milligrams of gallic acid equivalents per gram of dry weight (mg GAE/g dw).

#### 2.9.2. Total Anthocyanin Content

The TAC of the extracts was analyzed by the pH differential method [11]. The extracts were diluted with sodium acetate buffer (0.4 M, pH 4.5) and potassium chloride buffer (0.025 M, pH 1.0) to relevant spectrophotometric ranges (0.4–0.6). Next, the absorbance was read at 515 nm and 700 nm using an Eon BioTek spectrophotometer (Izasa, Barcelona, Spain). The TAC was expressed as milligrams of malvidin 3-*O*-glucoside equivalents per gram of dry weight (mg Mv3G Eq/g dw). The molar absorbance of Mv3G (493.44 g/mol) used was 28000 L/mol × cm.

#### 2.9.3. Total Flavanol Content

The TFC of extracts was estimated by the DMACA method [38]. Briefly, the samples (0.1 mL) were mixed with 0.5 mL of DMACA solution (0.1% 1 N HCl in MetOH) and allowed to react at room temperature for 10 min under protection from light exposure. The absorbance was then read at 640 nm using an Eon BioTek spectrophotometer (Izasa, Barcelona, Spain). Different Cat concentrations between 5–100 mg/L were used to construct the calibration curve. TFC was expressed as milligrams of (+)-Cat equivalents per gram of dry weight (mg Cat Eq/g dw).

#### 2.9.4. HPLC-DAD Analysis of Phenolic Compounds

Polyphenol separation was achieved using a ZORBAX Eclipse XDB-C18 (150 mm × 2.1 mm i.d., five-µm particle size) as the chromatographic column (Agilent Technologies, Palo Alto, CA, USA) equipped with a Narrow-Bore guard column (2.1 mm × 12.5 mm, 5 µm particle size). The mobile phase was water:acetic acid (95:5, *v*:*v*) (A) and acetonitrile:acetic acid (95:5, *v*:*v*) (B) in gradient mode as follows: initial conditions 0% B; 0–30% B, 0–18 min; 30–100% B, 18–19 min; 100% B isocratic, 19–20 min; 100–0% B, 20–21 min. A post-run of six minutes was required for column re-equilibration. The flow rate was set at 0.5 mL/min, and the injection volume was 10 µL for all of the runs. Before the injection, all of the samples were diluted 1:1 in mobile phase A.

Identification and quantification of the phenolic compounds of interest was achieved with a UV/Vis photodiode array detector (1260 Infinity, Agilent Technologies, Palo Alto, CA, USA). Chromatograms were recorded from 200 nm to 600 nm. Flavanols were detected at 280 nm [3,14], p-coumaric acid was detected at 290 nm, hydroxycinnamic acids and stilbenes were detected at 320 nm [3,14], flavonols were detected at 340 nm, and anthocyanins were detected at 520 nm [3,20]. When standard compounds were not available, flavanols were quantified as Cat equivalents, hydroxycinnamic acids were quantified as Chl equivalents, flavonols were quantified as Rut equivalents, and anthocyanins were quantified as Mv3G equivalents. The results were expressed as milligrams of equivalents per kilogram of dry weight (mg Eq/kg dw).

#### 2.9.5. HPLC-DAD Method Validation

Calibration curves, linearity, intraday variability (precision), interday variability (reproducibility), detection limits, and quantification limits were calculated in mobile phase A spiked with polyphenol standards (Table S1). The peak areas of various concentrations of standards were used to construct the calibration curves. The method’s precision was calculated as the relative standard deviation (% RSD) of the concentration in a triplicate analysis of three different spiked samples (50 µg/mL, 25 µg/mL, and one µg/mL). Method reproducibility was calculated as the relative standard deviation (% RSD) of three different standard compound concentrations (50 µg/mL, 25 µg/mL, and one µg/mL) analyzed in triplicate over three consecutive days. Sensitivity was evaluated by determining the limits of detection (LOD) and quantification (LOQ), respectively, which were defined as the concentrations corresponding to threefold and 10-fold of the signal-to-noise ratio.

### 2.10. Statistics

The results of the RSM design were analyzed using Design-expert 9.0.6 software (Trial version, Stat-Ease Inc., Minneapolis, MN, USA). SPSS 19 software (SPSS Inc., Chicago, IL, USA) was used for all of the other statistical analysis. All of the experiments were performed in triplicate; the statistical significance was evaluated using one-way ANOVA or Student’s *t*-test, and *p*-values less than *p* < 0.05 were considered to be statistically significant.

## 3. Results and Discussion

To quantify and accurately characterize the polyphenol content of food polyphenols, it is necessary to optimize the solid–liquid extraction process in order to obtain the highest polyphenol yield. Importantly, only those compounds that are extracted will be quantified. In this sense, the analysis of grape pulp is of key relevance, because this part of the fruit not only contains bioactive compounds such as hydroxycinnamic acids, but is also the predominate edible part of grapes [5,6,30,31]. However, to date, no specific methods for the extraction of whole red grape phenolics exist. Some factors, such as LSR, temperature, MetOH proportion, type of solvent, and time of extraction can greatly affect the polyphenol extraction yield from fruit matrixes [25,27]. Thus, it is essential to study the effect of these factors, both independently and combined, on the polyphenol extraction process. In this study, a polyphenol extraction method was optimized using the RSM for OG from *Vitis vinifera*. OG, CG, and PG, were analyzed whole (including seeds, skins, and pulp). The selection of OG was due to its higher phenolic content.

### 3.1. Single-Factor Studies

**Figure 1:**
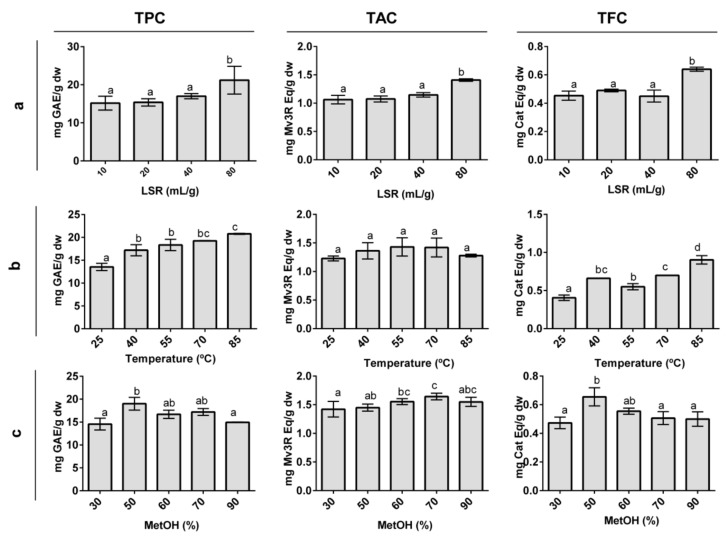
Effect of different liquid-to-solid ratios (**a**), temperatures (**b**), and methanol proportions (**c**) on total phenolic (TPC), total anthocyanin (TAC), and total flavanol (TFC) content in organic whole red Grenache grapes. The results are expressed as mg of phenolic equivalents per gram of dry weight (mg Eq/g dw) ± SD (n = 3). Letters represent significant differences (One-way ANOVA; *p* < 0.05). Abbreviations: liquid-to-solid ratio (LSR), methanol (MetOH), gallic acid equivalents (GAE), malvidin-3-*O*-rutinoside (Mv3R), and catechin (Cat).

TPC, TAC, and TFC were chosen to evaluate the effect of LSR, extraction temperature, and methanol proportion on the extractability of grape phenolic compounds (Figure 1), as they give a global view of the extractability of the major phenolic families found in grapes [2,3].

#### 3.1.1. Effect of Liquid-to-Solid Ratio

Four LSRs between 10–80 mL/g were chosen to evaluate the effect of the LSR on the polyphenol extraction yield and fix the optimal LSR. No higher LSRs were evaluated, as an increase in this variable increases production costs and might lead to the lack of detection of phenolic compounds present in low concentrations in grapes. The LSR showed a positive effect on the extraction of TPC, TAC, and TFC (Figure 1A), reaching higher yields when the LSR increased. Despite this, only the LSR of 80 mL/g showed a significant difference with other LSRs. Our results are in concordance with those from grape seed [39], black currants [40], and *Inga edulis* leaves [25] where the LSR effect was evaluated, and higher yields were obtained with a higher LSR. Moreover, our results are in agreement with the awareness that working at a low LSR can cause saturation problems [41]. Thus, a ratio of 80 mL/g was selected for the rest of the study.

#### 3.1.2. Effect of Temperature

Five different temperatures, ranging between 25–85 °C, were selected to study the effect of temperature on the polyphenol extraction yield (Figure 1B). The TAC was not statistically influenced by the extraction temperature, although temperatures between 40–70 °C appeared to show the highest anthocyanin yield. TAC values at 85 °C decreased slightly in comparison with the other extraction temperatures. This result seems plausible, given the thermosensitivity of anthocyanins. Indeed, anthocyanin degradation at similar and even lower temperatures was reported for black currants [40]. The TPC and TFC responded in a significant positive manner to temperature. Some studies have shown temperatures similar to 85 °C to be optimal for extracting polyphenols in grape cranes [19,20] and seeds [39]. However, other studies have reported polyphenol degradation when temperatures higher than 50 °C were used [26,27]. Temperatures lower than 40 °C have been reported to have a negative effect on polyphenol extraction [20,27,42]. The observed effect of the temperature can be explained by an increasing temperature modifying solvent properties, weakening polyphenol interactions with cell compounds, and compromising cell wall integrity, leading to an increase of polyphenol transference to the solvent [27,42]. Therefore, 40 °C, 65 °C, and 85 °C were selected as the low, medium, and high temperature values for the RSM, respectively.

#### 3.1.3. Effect of Methanol Proportion

Five different MetOH proportions ranging between 30–90% were chosen to study the effect of the percentage of solvent on the polyphenol extraction yield (Figure 1C). The TPC presented a higher extraction yield between 50–70% of MetOH, showing the highest extraction at 50% MetOH. Other studies have reported similar concentrations of solvent to be optimal for the extraction of TPC, as well as a decreasing TPC tendency when solvent concentrations increased [19,27,40]. The addition of water in organic extraction solvents promotes fruit particle swelling, increasing the contact area between the solvent and the fruit particle. This facilitates solvent penetration into the fruit particles, which enhances the extraction rate of phenolic compounds [43,44]. The TAC values showed significant differences between different percentages of MetOH and were higher at 70% MetOH. Indeed, anthocyanins are extracted at high organic solvent concentrations in *Euterpe oleracea* [18] and *Euterpe edulis* [45] fruits. The TFC showed a similar tendency as TPC, and concentrations between 50–60% MetOH resulted in the highest TFC. The extraction of apple polyphenols, which includes high quantities of flavanols, reported an optimal percentage of MetOH at approximately 60% [46]. The lowest TAC, TPC, and TFC values were obtained at 30% MetOH, clearly indicating that low MetOH proportions did not extract all of the polyphenols that were present in OG. Additionally, 90% MetOH produced a decrease in the extraction of TPC and TFC compared to 50% MetOH. Consistent with these findings, low MetOH percentages, as well as percentages of approximately 100%, are inefficient for the extraction of polyphenols [47]. Therefore, 40%, 60%, and 80% were selected as the low, medium, and high values of MetOH in the RSM study.

### 3.2. Surface Response Results

RSM was used to optimize the polyphenol extraction procedure. MetOH proportions ranging from 40% to 80% (*X_i_*) and temperatures ranging from 40 °C to 85 °C (*X_j_*) were selected based on the single factor study results. The previously optimized LSR of 80 mL/g and extraction time of 30 min were fixed throughout the RSM procedure. The selection of the extraction time was selected according to other studies in the literature [25,26,47]. The face-centered design setting of the independent variables using RSM and the experimental values of hydroxycinnamic acids (HCA), flavanols, anthocyanins, stilbenes, and flavonols quantified by HPLC-DAD are shown in Table 1. Individual compounds quantified by the HPLC-DAD method were used as dependent variables in the RSM design. The individual responses of anthocyanins, flavanols, flavonols, and HCA were demonstrated to depend on the extraction variables, whereas no Rvt, Chl, and pCou were detected in any of the extractions. Compounds not responding to the independent variables were omitted from the model.

#### 3.2.1. Fitting the Model

The experimental data in Table 1 were used to determine the coefficients of the second-order polynomial equation (Equation (1)). Several compounds generated a significant extraction model, implying that at least one of the extraction variables can explain the variation in the response variables (Table 2). Furthermore, most of the R^2^ values of the parameters were greater than 0.80, meaning that the model accurately represented the experimental data. All of the models that were generated were highly significant, as the p-values ranged between 0.018–0.001. In addition, the lack of fit was not significant (*p* > 0.05), which indicated that the model could adequately fit the data, thus further validating the model. On the other hand, when Cy3R, Mv3G, malvidin-3-*O*-glucoside equivalent 1 (Mv3G Eq1), malvidin-3-*O*-glucoside equivalent 2 (Mv3G Eq2), malvidin-3-*O*-glucoside equivalent 4 (Mv3G Eq4), and chlorogenic acid equivalent 1 (Chl Eq1) were analyzed, the predicted R^2^ was negative (data not shown), implying that the model was not a correct predictor for the response, and the responses were not influenced by either the percentage of MetOH or the temperatures. Therefore, these compounds were omitted in the optimization process. Additionally, the results showed that Cat and Ecat responses had a better fit to a linear model, which is in agreement with the studies of Pinelo et al., who also reported linear behaviors in phenolic extractions through RSM [21].

When RSM designs are applied, it is important to understand that the effect of a factor in the mass transfer process is not always clear. The chemical characteristics of the solvent and the diversity of structures and composition of natural products allow each combination of material and solvent system to show different behaviors that cannot be predicted [21].

#### 3.2.2. Combined Effect of Temperature and Methanol

**Figure 2:**
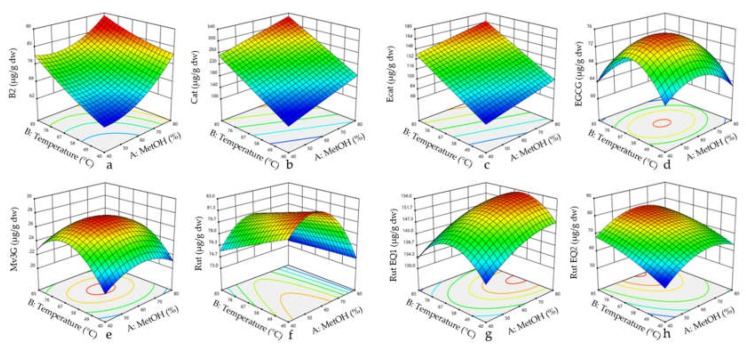
Response surface plots for procyanidin dimer B2 (**a**), catechin (**b**), epicatechin (**c**), EGCG (**d**), malvidin-3-*O*-glucoside equivalent 3 (**e**), rutin (**f**), rutin equivalent 1 (**g**), and rutin equivalent 2 (**h**) regarding the function of methanol proportion and temperature. Abbreviations: Cat, catechin; Ecat, epicatechin; EGCG, (−)-epigallocatechin gallate; EQ, equivalent; Rut, rutin; Mv3G, malvidin-3-*O*-glucoside; and MetOH, methanol.

The regression coefficients of the model (Equation (1)) for the studied compounds obtained by the multiple linear regression are presented in Table 2. The dependent variables (individual responses of the specific polyphenols studied) allowed a direct interpretation of the effect of the independent variables (MetOH proportion and extraction temperature). The visualization of the statistical significance of the independent variables on the dependent variables was facilitated by generating surface contour plots (Figure 2).

Individual phenolic compounds were affected differently by the extraction temperature and methanol concentration (Table 2). The temperature produced a positive linear effect on B2, Cat, Ecat, and Rut Eq2 extraction, and MetOH had a positive linear effect on Mv3G Eq3, Rut, Rut Eq1, and Rut Eq2. Positive linear effects of temperature were also reported for several phenolics in cherry, grape seeds and *Inga edulis* leaves [25,30,47]. A negative quadratic effect of temperature was described for Rut Eq1, EGCG and Mv3G Eq3, while a negative quadratic effect of MetOH was obtained for EGGG, Mv3G Eq3, Rut and Rut Eq2 (Figure 2). This means that the yields of these compounds increase when the temperature and/or MetOH proportion increases up to a certain point, after which they begin to decrease. Indeed, negative quadratic effects of both solvent concentration and temperature are very common in RSM studies, and have been reported in the extraction of polyphenols from vegetal matrixes such as sour cherries [47], *Inga edulis* leaves [25], and *Euterpe oleracea* fruits [18]. A negative linear effect of methanol was found for B2, whereas no linear or quadratic significant effect was reported for Cat and Ecat, suggesting that in the range of the MetOH concentrations that were evaluated, the compounds were not differently extracted. These results are in disagreement with studies where Cat and total flavanols showed positive linear and negative quadratic effects in sour cherries [47] and *Inga edulis* leaves [25]. This may be due to a more restrictive MetOH range and the different plant matrix. Nevertheless, there are examples of linear behaviors in the literature [21]. Moreover, a positive quadratic effect of methanol was observed for B2, meaning a B2 extraction increase with an increase in MetOH, theoretically up to 100%. Some studies have reported linear and quadratic effects with total anthocyanins, but the MetOH range that was used in those studies was less selective than that used in this study [18,47].

In our study, none of the analyzed parameters presented an interaction between MetOH and temperature. Although some studies do report these types of interactions [20,45], most of the studies do not [18,25,47].

#### 3.2.3. Method Validation

The combination extraction variables at the highest desirability (0.686) were a temperature of 72 °C and 65% MetOH (Table 3). To validate the model, three extractions were performed under those predicted conditions. HCAs, flavanols, anthocyanins, and flavonols were quantified by HPLC-DAD and compared with the model-predicted values. The results showed no significant differences between the predicted and the experimental values (Table 3). Therefore, the extraction temperature and methanol proportion were fixed at 72 °C and 65%, respectively, throughout the rest of the study. The optimized extraction temperature of 72 °C is very similar to that reported for sour cherries [47]. However, higher optimal extraction temperatures were reported for the extraction of antioxidants [20] and TPC [19] from grape cranes. Additionally, lower temperatures were also found to be optimal in the extraction of antioxidants from grape stalks [37]. In agreement with our optimized methanol proportion, Karacabey et al. found similar ethanol percentages to be optimal in the extraction of phenolics from grape cranes [19]. Furthermore, other plant matrixes show the optimal extraction of phenolic compounds at similar organic solvent percentages [26,27]. Importantly, the differences between the optimal extraction conditions found in this study and the optimal conditions reported for other grape parts [19,20] reflect the need for optimization studies for each food matrix. In agreement with this, Karvela et al. demonstrated that the optimized conditions for the extraction of grape seeds from varied among different grape varieties [36].

### 3.3. Effect of Time on Polyphenol Extraction

To further optimize the polyphenol extraction method, a kinetic study was performed. Seven extraction times ranging from 0 to 120 min were selected to perform the study. The LSR was fixed at 80 mL/g, the temperature at 72 °C, and MetOH at 65%. The effect of time was evaluated by analyzing the extraction rate of TPC, TAC, and TFC, which gives a general view of the effect of time on the extraction of the major phenolic families present in grapes [2,3].

**Figure 3:**
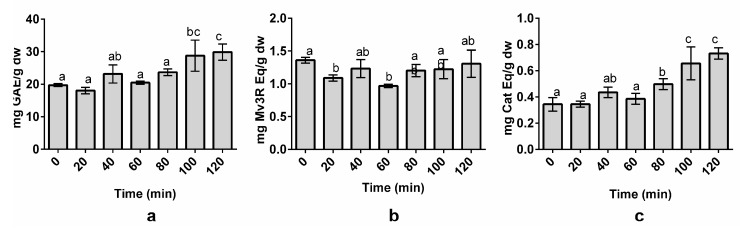
Effect of time on total phenolic content (TPC) (**a**), total anthocyanin content (TAC) (**b**), and total flavanol content (TFC) (**c**) of organic whole red Grenache grapes. The results are expressed as mg of phenolic equivalents per gram of dry weight (mg Eq/g dw) ± SD (n = 3). Letters represent significant differences (one-way ANOVA; *p* < 0.05). Abbreviations: gallic acid equivalents (GAE), malvidin-3-*O*-rutinoside (Mv3R), and catechin (Cat).

TPC and TFC (Figure 3A,C) displayed a significant sensitivity to the effect of time during the extraction process, indicating that higher extraction times allow for increased TPC and TFC yields. Indeed, longer extraction times usually resulted in an increase of polyphenol yields [27]. The highest TAC (Figure 3B) reported for the anthocyanin yield was obtained at an extraction time of 0 min. This can be explained because anthocyanins are thermosensitive, and high temperatures can cause anthocyanin degradation over time [40]. Despite this, the TAC did not show significant differences with higher extraction times (80–120 min). Therefore, the extraction time was fixed at 100 min throughout the rest of the experiment. Yilmaz et al. reported an extraction time of 80–100 min to obtain the maximum TAC and TPC values from sour cherry [47]. Armendola et al. reported an extraction time of 120 min to obtain the highest yield of polyphenols from grape marc [22]. The need for such a high extraction time may be a consequence of the high polyphenol content found in grapes or the food matrix itself. In fact, it is known that polyphenols are bonded to some components of the food matrix [23]. Exposure to high temperatures and optimal MetOH concentrations over certain periods of time allows for the solubilization of these polyphenols due to the weakening of the cell wall and polyphenol–component interactions [27,42]. This leads to polyphenol transference into the solvent, thereby increasing the polyphenol yield in the extracts [27].

### 3.4. Multi-Step Extractions

**Figure 4:**
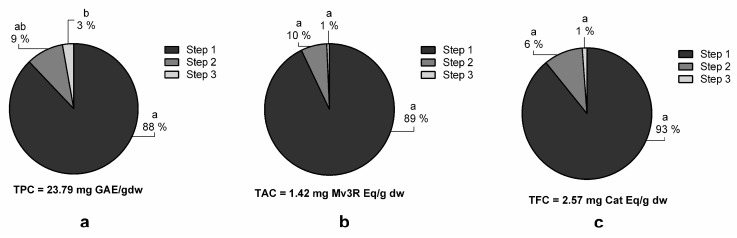
Effect of different extraction steps on total phenolic (TPC) (**a**), total anthocyanin (TAC) (**b**), and total flavanol (TFC) (**c**) content of organic whole red Grenache grapes. The total amount after three consecutive extractions is expressed as mg of phenolic equivalents per gram of dry weight (mg Eq/g dw) ± SD (n = 3). Letters represent significant differences (one-way ANOVA; *p* < 0.05). Abbreviations: gallic acid equivalents (GAE), malvidin-3-*O*-rutinoside (Mv3R) and catechin (Cat).

To evaluate the effect that multi-step extractions have on polyphenol extraction yields, three consecutive extractions were performed under the optimized conditions (LSR 80 mL/g, temperature 72 °C, 65% MetOH, and 100-minute extraction time) (Figure 4). The statistical analysis shows that each extraction step statistically increases the TPC (Figure 4A). Specifically, the TPC increased from 25.33 ± 1.77 in the first extraction to 28.93 ± 1.74 mg GAE/g dw in the third extraction. However, no significant increases were achieved for TAC or TFC (Figure 4B,C, respectively) between the extraction steps. Similarly, Mané et al. reported that flavanols, phenolic acids, and anthocyanins in grape skins, seeds, and pulp were predominantly extracted in the first extraction step [30]. From an economical and practical point of view, it was decided that only one extraction step was the optimal condition for grape polyphenol extraction, as TPC only increased by 3 mg GAE/g dw in the third extraction step compared to the first.

### 3.5. Methanol–Ethanol Comparison

**Figure 5:**
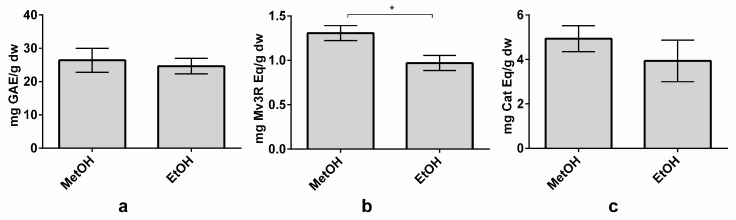
Effect of methanol (MetOH) and ethanol (EtOH) on total phenolic content (TPC) (**a**), total anthocyanin content (TAC) (**b**), and total flavanol content (TFC) (**b**) on organic whole red Grenache grapes. Results are expressed as mg of phenolic equivalents per gram of dry weight (mg Eq/g dw) ± SD (n = 3). * *p* < 0.05. Abbreviations: gallic acid equivalents (GAE), malvidin-3-*O*-rutinoside (Mv3R) and catechin (Cat).

Due to the potential application of ethanol in the food industry to obtain extracts for commercialization, a methanol-based extraction and an ethanol-based extraction were carried out to evaluate whether both solvents achieved the same results. Extractions were performed under the optimized conditions of LSR of 80 mL/g, MetOH or EtOH concentrations of 65% (1% formic acid), a temperature of 72 °C, and an extraction time of 100 min. TPC and TFC did not present significant differences between the methanol and ethanol extractions, although methanol extraction achieved slightly higher values of TPC and TFC (Figure 5A,C). These results are in agreement with those obtained by Tabaraki et al., who found only small differences between solvents during the extraction process in grape seeds [42]. However, TAC was reported to be statistically higher in MetOH (1.31 ± 0.08 mg Mv3G Eq/g dw) than in EtOH (0.97 ± 0.08 mg Mv3G Eq/g dw) extraction (Figure 5B). In fact, the anthocyanin yield decreased by nearly 26% in the ethanol extraction. This result is in accordance with the study by Metivier et al., where the grape anthocyanin content was 20% higher in the methanol extraction [48]. Indeed, methanol extractions are usually more efficient, obtaining higher polyphenol yields than ethanol extractions [24,49]. Even though some differences are reported in the TAC, global differences between the two solvents are minor. Therefore, this method could be used in the food industry. Nevertheless, more studies are needed to adapt it for large-scale use.

### 3.6. Full Characterization of Whole Red Grapes

The profile and content of phenolic compounds in grapes is known to be affected by many factors [3,14,15], including cultivation conditions [15] and grape variety [4,14,17]. OG, CG, and PG underwent extraction at the optimized conditions of 80 mL/g, 65% MetOH, and 72 °C for 100 min in one single extraction step to assess the effect of grape variety and culture system on the phenolic profile of whole red grapes. The cultivation methodology (organic or conventional) effect was evaluated by comparing OG and CG, while the grape variety was evaluated by comparing PG with CG. When individual polyphenol responses were analyzed by the HPLC-DAD method (Table 4), OG, CG, and PG presented Mv3G and Cat as the main anthocyanin and flavanols, respectively. Several Vitis vinefera varieties reported Cat as the most concentrated flavanol found in both seeds and skins [3,4,17], and Mv3G as the main anthocyanin found in skins [3,29]. Indeed, Mv3G was the most abundant phenolic in all of the grapes, followed by Chl Eq1 and Cat in OG and CG, and by Cat and Ecat in PG. Grapes have reportedly high concentrations of HCA in skins, and also in the pulp [5], which could explain the abundance of Chl EQ1. In all three grape varieties, flavonols were detected in lower quantities than anthocyanins and flavanols, which has been previously described in several Vitis vinifera varieties [4,29]. Studies with red grape skins have reported resveratrol at concentrations of approximately 0.6–25 mg/g skins [17]. Given the low concentration of resveratrol in skins and the use of whole red grapes in this study, the concentration of resveratrol in the extracts might be extremely low, thus leading to its lack of detection.

OG showed the highest values of TPC, TAC, and TFC, whilst PG showed the lowest. The individual polyphenol responses were in agreement with the previously determined total phenolic and total anthocyanin values (Table 4). Importantly, OG presented higher concentrations of some individual anthocyanins and lower concentrations for some flavonols than CG. These results are also in agreement with those found by Mulero et al. in organic and conventional Mourvèdre grapes [15]. Indeed, studies investigating differences between organic and conventional foods have reported higher contents of polyphenols, although not always statistically significant, in organic foods [15,50]. This is in agreement with phenolic compounds being stress metabolites synthetized under adverse conditions [12]. The PG polyphenolic profile presented fewer polyphenolic compounds, in particular anthocyanins, as well as a reduction in their levels when compared to Grenache CG. Indeed, large differences in skin anthocyanin contents between different grape varieties have been described by Kammerer et al. and Cantos et al. [3,14].

The number of studies evaluating the phenolic profile in whole red grapes is currently limited [32,33]. By applying our method, higher concentrations of relevant phenolic constituents (i.e., catechin, epicatechin, and malvidin-3-*O*-glucoside) were found in our grape varieties than in the study by Lingua et al., in which whole red grapes were analyzed [33]. Grape pulp contains phenolic compounds, especially hydroxycinnamic acids [5,6,30]. In this sense, Mulero et al. reported total hydroxycinnamic acid at concentrations as high as 22.81 ± 0.24 mg/kg of whole fresh weight, when only skins were analyzed [15]. In our study, which also includes the pulp, the extraction of CHL EQ1 resulted in clearly superior quantities in the Grenache varieties (OG and CG). In summary, this study suggests that the extraction of phenolic compounds from whole red grapes provides a higher extraction of phenolic compounds, and this is essential to be able to correlate the health effects associated with the consumption of grapes and the phenolic compounds that are responsible for these effects.

## 4. Conclusions

Our study reported the optimal conditions for the extraction of the most representative phenolics from whole red grapes using a MetOH proportion of 65% (1% formic acid), a temperature of 72 °C, an LSR of 80 mL/g, and an extraction time of 100 min in a one-step extraction. These conditions differed from previous methodologies for specific grape parts (i.e., skins or seeds), which demonstrated the need for a specific extraction methodology when using whole red grapes. To our knowledge, no similar methodologies have been developed for the extraction of whole red grapes. This work suggests that the analysis of whole grape phenolics, including pulp, will result in a higher quantification of phenolic compounds. Moreover, if adapted to a larger scale, ethanol could be used to produce phenolic-rich extracts for commercialization. This study demonstrates that organic cultivation systems affect the phenolic compound profile of red Grenache grapes, promoting a higher content of phenolic compounds, especially anthocyanins.

## Figures and Tables

**Table 1:** Face-centered design setting of the independent variables and experimental results of flavanols, flavonols, hydroxycinnamic acids, stilbenes, and anthocyanins quantified by HPLC-DAD of organic whole red Grenache grapes.

Run ^1^	MetOH (%)	T (°C)	B2	Cat	Ecat	EGCG	Rut	Rut EQ1	Rut EQ2	Chl	Chl EQ1	pCou	Rvt	Cy3R	Mv3G	Mv3G EQ1	Mv3G EQ2	Mv3G EQ3	Mv3G EQ4
1	40	40	60.66	136.93	74.27	63.95	81.08	133.00	56.47	n.d.	825.48	n.d.	n.d.	135.47	1159.52	104.39	37.57	20.26	181.29
2	80	40	79.63	177.73	100.23	62.89	75.20	146.23	65.66	n.d.	796.71	n.d.	n.d.	151.31	1253.41	119.17	43.96	21.19	216.30
3	40	85	79.82	230.51	124.36	63.03	74.90	135.20	67.32	n.d.	760.54	n.d.	n.d.	144.71	1194.49	111.79	33.17	22.99	188.68
4	80	85	90.47	363.22	180.12	60.74	73.44	146.81	73.75	n.d.	925.75	n.d.	n.d.	139.57	1209.93	113.83	36.78	22.74	201.51
5	40	65	72.29	168.54	128.96	73.44	78.05	143.62	63.99	n.d.	853.20	n.d.	n.d.	142.10	1284.35	133.39	32.91	25.37	210.41
6	80	65	83.00	251.54	124.24	69.12	73.76	153.93	70.50	n.d.	838.44	n.d.	n.d.	133.90	1163.91	113.00	36.41	24.52	200.34
7	60	40	68.95	158.76	78.99	69.09	81.25	147.06	63.77	n.d.	860.99	n.d.	n.d.	145.19	1176.84	119.36	36.24	22.90	198.69
8	60	85	78.02	350.76	146.46	67.14	80.61	138.68	80.25	n.d.	826.50	n.d.	n.d.	141.28	1276.78	117.86	42.36	23.53	214.59
9	60	65	70.79	208.79	128.10	72.67	81.34	150.34	75.91	n.d.	743.96	n.d.	n.d.	134.92	1175.77	120.11	38.09	26.86	191.47
10	60	65	74.60	191.36	122.34	73.67	82.88	151.35	76.61	n.d.	758.96	n.d.	n.d.	140.03	1244.41	136.07	40.21	28.83	205.79
11	60	65	72.44	221.15	129.73	76.89	78.79	156.21	77.25	n.d.	799.85	n.d.	n.d.	132.07	1271.07	117.57	34.31	26.69	216.11

Abbreviations: Methanol (MetOH); temperature (T); not detected (n.d.); procyanidin dimer B2 (B2); (+)-catechin (Cat); (−)-epicatechin (Ecat); (−)-epigallocatechin gallate (EGCG); rutin (Rut); rutin equivalent 1 (Rut Eq1); rutin equivalent 2 (Rut Eq2); chlorogenic acid (Chl); chlorogenic acid equivalent 1(Chl Eq1); p-coumaric acid (pCou); resveratrol (Rvt); cyanidin-3-rutinoside (Cy3R); malvidin-3-*O*-glucoside (Mv3G); malvidin-3-*O*-glucoside equivalent 1 (Mv3G Eq1); malvidin-3-*O*-glucoside equivalent 2 (Mv3G Eq2); malvidin-3-*O*-glucoside equivalent 3 (Mv3G Eq3); and malvidin-3-*O*-glucoside equivalent 4 (Mv3G Eq4); high-performance liquid chromatography with a diode array detector (HPLC-DAD). Results are expressed as milligrams of phenolic compound per kilogram of dry weight (mg /kg dw). ^1^ All extractions were carried out for 30 min, with 500 rpm agitation.

**Table 2:** Analysis of the variance and regression coefficients of the predicted model for the response variables of organic whole red Grenache grapes.

Model Parameters	Regression Coefficient	B2	Cat	Ecat	EGCG	Mv3G Eq3	Rut	Rut Eq1	Rut Eq2
Intercept	β_0_	66.132	−98.331	−10.753	−20.457	−23.042	55.243	33.532	−45.193
Linear									
MetOH	β_1_	−0.742 *	1.912	0.642	1.293	0.624 ^#^	1.228 *	1.307 *	2.593 *
T	β_2_	0.285 *	3.217 *	1.475 *	1.914	0.973	−0.193 ^#^	2.314	0.926 *
Interaction									
MetOH × T	β_12_	−4.775 × 10^−3^	-	-	−7.870 × 10^−4^	−6.990 × 10^−4^	2.422 × 10^−3^	−9.810 × 10^−4^	−1.579 × 10^−3^
Quadratic									
MetOH × MetOH	β_11_	1.151 × 10^−2^ *	-	-	−1.089 × 10^−2^ *	−4.842 × 10^−3^ *	−1.232 × 10^−2^ *	−7.934 × 10^−3^	−1.924 × 10^−2^ *
T × T	B_22_	2.328 × 10^−3^	-	-	−1.523 × 10^−2^ *	−7.156 × 10^−3^ *	−1.250 × 10^−4^	−1.837 × 10^−2^ *	−4.548 × 10^−3^
R^2^		0.961	0.847	0.874	0.9456	0.9283	0.8935	0.9037	0.99553
Adjusted R^2^		0.922	0.809	0.843	0.8912	0.8567	0.7871	0.8074	0.9105
*p*-value		0.002	<0.001	<0.001	0.0035	0.007	0.018	0.014	0.002
F-value		24.63	22.12	27.82	17.38	12.95	8.39	9.38	21.36
Lack of fit		0.442	0.173	0.080	0.776	0.742	0.821	0.495	0.056

Abbreviations: Methanol (MetOH); temperature (T); determination coefficient (R^2^); procyanidin dimer B2 (B2); (+)-catechin (Cat); (−)-epicatechin (Ecat); (−)-epigallocatechin gallate (EGCG); malvidin-3-*O*-glucoside equivalent 3 (MV3G Eq3); rutin (Rut); rutin equivalent 1 (Rut Eq1); and rutin equivalent 2 (Rut Eq2). Differences between groups determined by ANOVA * *p* < 0.05, ^#^ *p* < 0.01.

**Table 3:** Overall optimal values of extraction parameters for flavanols, anthocyanins, flavonols, and phenolic acids of organic whole red Grenache grapes.

Extraction Variables ^1^			Parameter	Predicted	Experimental		
T (°C)	MetOH (%)	Desirability					
72	65	0.686	B2	76.70	77.73	±	1.17
			Cat	257.57	264.44	±	17.64
			Ecat	137.16	153.03	±	17.03
			EGCG	72.74	73.71	±	3.25
			Cy3R	136.56	144.59	±	1.73
			Mv3G	1233.23	1363.78	±	7.49
			Mv3G Eq1	118.77	131.95	±	8.61
			Mv3G Eq2	37.64	38.23	±	1.45
			Mv3G Eq3	26.72	26.33	±	1.98
			Mv3G Eq4	202.23	219.43	±	6.19
			Rut	79.80	82.75	±	1.05
			Rut Eq1	151.80	151.53	±	1.47
			Rut Eq2	77.80	76.14	±	3.37
			Chl Eq1	828.07	888.50	±	4.73

Abbreviations: Temperature (T); methanol (MetOH); procyanidin dimer B2 (B2); (+)-catechin (Cat); (−)-epicatechin (Ecat); (−)-epigallocatechin gallate (EGCG); cyanidin-3-rutinoside (Cy3R); malvidin-3-*O*-glucoside (Mv3G); malvidin-3-*O*-glucoside equivalent (Mv3G Eq); rutin (Rut); rutin equivalent (Rut Eq); and chlorogenic acid equivalent (Chl Eq). Results are expressed as mg of phenolic component per kilogram of dry weight (mg Eq/g dw) ± SD (n = 3). No significant differences were determined (Student’s *t*-test; *p* < 0.05). ^1^ All extractions were carried out for 30 min, with 500 rpm agitation.

**Table 4:** Generic polyphenol variables and individual polyphenol contents analyzed by HPLC-DAD of conventional whole Grenache grapes (CG), red organic whole red Grenache grapes (OG), and whole red Peruvian Red Globe grapes (PG).

	CG	OG	PG
TPC ^1^	23.98 ± 2.60	26.37 ± 3.60	17.18 ± 1.87 *
TAC ^1^	0.98 ± 0.11	1.31 ± 0.08*	0.71 ± 0.05 *
TFC ^1^	4.42 ± 0.92	4.93 ± 0.58	3.68 ± 0.20
Cat	519.51 ± 179.55	382.30 ± 90.06	255.82 ± 68.72
B2	139.29 ± 43.04	95.88 ± 10.09	39.15 ± 4.14 *
EGCG	N.D.	99.47 ± 6.45	N.D.
Ecat	276.45 ± 98.22	162.42 ± 46.17	241.48 ± 14.81
Chl EQ1	797.05	853.16 ± 57.44	176.31 ± 6.69 *
Chl	N.D.	N.D.	N.D.
Rvt	N.D.	N.D.	N.D.
pCou	N.D.	N.D.	N.D.
Mv3G EQ1	34.38 ± 11.23	133.81 ± 10.29 *	N.D.
Cy3R	79.57 ± 8.81	159.83 ± 6.20 *	161.31 ± 11.97 *
Mv3G	1116.80 ± 67.21	1534.89 125.26 *	887.14 ± 57.14 *
Mv3G EQ2	36.95 ± 10.36	53.36 ± 15.01	N.D.
Mv3G EQ3	69.58 ± 1.50	79.52 ± 10.42	N.D.
Mv3G EQ4	198.09 ± 28.08	199.30 ± 31.98	42.39 ± 5.22*
Rut	157.72 ± 20.31	117.13 ± 13.26 *	60.81 ± 11.6 *
Rut EQ1	318.48 ± 63.43	172.48 ± 13.57 *	116.17 ± 19.60 *
Rut EQ2	111.03 ± 21.27	82.49 ± 4.01	N.D.

Abbreviations: Total polyphenol content (TPC); total anthocyanin content (TAC); total flavanol content (TFC); not detected (n.d.); (+)-catechin (Cat); procyanidin dimer B2 (B2); (−)-epigallocatechin gallate (EGCG); (−)-epicatechin (Ecat); chlorogenic acid equivalent 1 (Chl Eq1); chlorogenic acid (Chl); resveratrol (Rvt); p-coumaric acid (pCou); malvidin-3-*O*-glucoside equivalent 1 (Mv3G Eq1); cyanidin-3-rutinoside (Cy3R); malvidin-3-*O*-glucoside (Mv3G); malvidin-3-*O*-glucoside equivalent 2 (Mv3G Eq2); malvidin-3-*O*-glucoside equivalent 3 (Mv3G Eq3); malvidin-3-*O*-glucoside equivalent 4 (Mv3G Eq4); rutin (Rut); rutin equivalent 1 (Rut Eq1); and rutin equivalent 2 (Rut Eq2). * represents significant differences with CG by Student’s *t*-test (*p* < 0.05). Results are expressed as mg of phenolic component per kilogram of dry weight ± SD (n = 3). ^1^ Generic polyphenol variables. The results are expressed as mg of phenolic component per gram of dry weight ± SD (n = 3).

## Supplementary Materials

The following are available online at http://www.mdpi.com/2072-6643/10/12/1931/s1, Table S1: Retention time (Rt), detection wavelength, calibration curves, determination coefficient (R^2^), linearity range, method precision, method reproducibility, limit of detection (LOD) and limit of quantification (LOQ) for phenolic compound quantification in spiked Milli-Q water:acetic acid (95:5 *v*:*v*) solution using HPLC-DAD.
