# Supplementary material for: Optimized Extraction by Response Surface Methodology Used for the Characterization and Quantification of Phenolic Compounds in Whole Red Grapes (Vitis vinifera)
Source: Nutrients. 2018 Dec 5;10(12):1931. doi: 10.3390/nu10121931 (PMC6316023; doi:10.3390/nu10121931)
Supplement: Supplementary file 1 [file nutrients-10-01931-s001.pdf]

Supplementary Table 1: Retention time (Rt), detection wavelength, calibration curves, determination coefficient (R^2), linearity range, method precision, method reproducibility, limit of detection (LOD) and limit of quantification (LOQ) for phenolic compound quantification in spiked Milli-Q water:acetic acid (95:5 v:v) solution using HPLC-DAD.

**Compound**	**Rt (min)**	**Detection wavelength (nm)**	**Calibration Curve**	**R^2**	**Linearity (nM)**	**Precision (% RSD, n=3)**			**Reproducibility (% RSD, n=3)**			**LOD (µM)**	**LOQ (µM)**
						**50 µg/mL**	**25 µg/mL**	**1 µg/mL**	**50 µg/mL**	**25 µg/mL**	**1 µg/mL**		
Cat	6.12	280	y=12.512x	0.9971	3.44 - 172.25	3.77	4.70	1.59	6.79	2.31	0.62	0.342	1.140
Chl	7.16	320	y=25.385x	0.9972	1.41 - 141.12	5.18	3.37	2.00	4.22	1.47	0.09	0.040	0.135
B2	7.40	280	y=37.814x	0.9905	0.17 - 86.43	9.88	5.86	2.03	2.92	5.43	1.45	0.005	0.015
EGCG	8.46	280	y=27.051x	0.9977	1.09 - 109.08	4.84	1.81	0.79	3.78	1.67	1.99	0.034	0.113
Ecat	9.27	280	y=13.466x	0.9904	1.72 - 172.25	9.49	5.38	9.20	5.14	0.76	1.08	0.089	0.296
Cy3R	10.35	520	y=51.985x	0.9948	0.17 - 83.96	5.73	6.10	5.11	4.58	0.22	4.16	0.003	0.010
pCou	10.69	290	y=150.890x	0.9960	0.61 - 304.58	5.87	3.35	1.06	4.06	1.01	3.85	0.003	0.010
Mv3R	11.71	520	y=46.252x	0.9940	0.20 - 101.33	5.88	6.85	5.84	4.21	0.31	5.56	0.006	0.021
Rut	13.51	340	y=27.135x	0.9965	0.16 - 81.90	5.98	3.42	2.41	4.49	1.33	0.19	0.003	0.010
Rvt	16.14	320	y=130.550x	0.9965	0.22 - 219.07	5.91	3.34	0.84	4.40	1.42	0.19	0.002	0.006

Abbreviations: (+)-catechin (Cat); chlorogenic acid (Chl); procyanidin dimer B2 (B2); (-)-epigallocatechin gallate (EGCG); (-)-epicatechin (Ecat); cyanidin-3-O-rutinoside (Cy3R); p-coumaric acid (pCou); malvidin-3-O-glucoside (MV3G); rutin (Rut) and resveratrol (Rvt).
